# Exploring a cherubism bone phenotype outside the craniofacial region

**DOI:** 10.1186/s13023-026-04413-3

**Published:** 2026-06-11

**Authors:** Anne Morice, Philippe Drabent, Sylvie Thomasseau, Aline Joly, Gérard Maruani, Marie Piketty, Séverine Brabant, Jeanne Amiel, Arnaud Picard, Natacha Kadlub, Amélie E. Coudert

**Affiliations:** 1 Service de chirurgie maxillo-faciale et plastique de la face, CHRU de Tours, Hôpital Clocheville, Centre de compétence MAFACE et CRANIOST, Tours, 37044 France; 2 Faculté de médecine de Tours, Université de Tours, Tours, France; 3 Pathology Department, Necker-Enfants Malades Hospital, APHP, Paris, France; 4 BIOSCAR, INSERM U1132, Université de Paris Cité, Hôpital Lariboisière, Paris, 75010 France; 5 Institut Necker Enfants-Malades, INSERM U1151 – CNRS UMR 8253, Université Paris Cité, Paris, France; 6 Service de Physiologie, Hôpital Necker - Enfants Malades and Hôpital Européen Georges Pompidou, Assistance Publique-Hôpitaux de Paris, Paris, 75015 France; 7 APHP, Necker Enfants Malades, Service des Explorations Fonctionnelles, Paris, 75015 France; 8 Département de Génétique, Hôpital Necker-Enfants Malades, Institut Imagine, Assistance Publique–Hôpitaux de Paris, Université Paris Cité, Paris, France; 9 APHP, Necker Enfants Malades, Service de Chirurgie Maxillo-faciale et Plastique, Paris, 75015 France; 10 APHP, CRMR des Malformations Rares de la Face et de la Cavité Buccale, Paris, 75015 France; 11 Laboratoire de Mécanique et de ses Interfaces, ENSTA-Institut Polytechnique de Paris, Palaiseau, France; 12 UFR de Médecine, Université de Paris Cité, Paris, 75006 France; 13 UFR d’Odontologie, Université de Paris Cité, Paris, 75006 France

**Keywords:** Cherubism, Bone density, Blood markers, NFATc1

## Abstract

Cherubism is a rare paediatric bone disease caused by gain-of-function mutations in the *SH3BP2* gene. This condition is characterized by osteolysis of the jaw bone, which can be sometimes massive, whereby bone is replaced by fibrous tissue containing osteoclast-like multinucleated giant cells. Recently, a patient with a severe cherubism was reported to have, in addition to the craniofacial cherubism features, a low bone mass phenotype. To determine whether this patient was the exception or the rule, the cherubism phenotype was explored in ten more patients outside the cranio-facial. For the first time, inflammatory and bone blood markers together with bone density and growth in height and weight were systematically assessed in relation to the radiological and NFATc1 location classifications. We observed normal weight and height in the patients, as well as a significant increase in the bone metabolism blood markers (especially CTx and P1NP), which was associated with the severity of the cherubism. Our analysis also highlights the need for more systematic assessments of cherubism patients to improve our understanding of the cherubism natural history.

**Supplementary Information:**

The online version contains supplementary material available at 10.1186/s13023-026-04413-3.

## Clinical trial number

Not applicable

**Supplementary Information:**

The online version contains supplementary material available at 10.1186/s13023-026-04413-3.

## Introduction

Cherubism (OMIM #118400) is a rare autosomal dominant genetic condition, belonging to the family of benign osteolytic tumours of the jaws [1]. To date, around 600 cases of cherubism have been reported in different ethnic groups. This condition is characterized by the progressive expansion of the cortical bone linked to the proliferation of giant cell granulomas. Diagnosis of cherubism diagnosis is based on clinical and radiological signs, histological features and genetic analysis. The condition primarily affects the mandible and/or the maxilla, typically bilaterally. The natural progression of cherubism can be described as follows: expansion from the age of two to puberty, followed by stabilisation and regression [2, 3]. Clinically, cherubism presents as a painless enlargement of the jawbone. Cherubism is a progressive condition characterized by varying degrees of severity, ranging from asymptomatic or mild cases to those that are very severe and potentially life-threatening ones [2]. The severity grading is based on the location and the extent of the bone lesions caused by cherubism. The most widely used classification system is based on the Raposo-Amaral & Motamedi classifications [1, 4, 5]. Depending on the severity, patients may exhibit mandibular and maxillary enlargement, dental malposition and, depending of the severity, upper airway and nasal obstruction, eye proptosis, and severe facial deformities [2, 3]. Histologically, the cherubism granulomas are composed of multinucleated giant cells (MGCs) and fibrous stroma [6]. The factors leading to the onset of cherubism are not fully understood. Although late revelation or reactivation of cherubism has been described in adults [4, 7], most of cherubism cases are revealed in infancy, supporting the hypothesis that tooth eruption and mastication forces are potential initiating factors. Current treatments range from observation to extensive surgery or chemotherapy, though the efficacy of each approach remains limited. Several drug therapies have been administered to patients with cherubism, yielding various outcomes (for review see [8]), but no standardized protocol has yet been established. This is why surgery is still widely recommended to reduce the size of the cherubism bone lesions.

Cherubism is mainly caused by point mutations in the exon 9 of the *SH3BP2* gene, which is located on chromosome 4p16.3. These mutations can be acquired by autosomal transmission or *de novo*. The *SH3BP2* gene encodes for a ubiquitous adaptor protein called SH3BP2 [9]. The penetrance and expressivity of the disease vary greatly with no correlation between genotype and phenotype [2]. In addition to the aesthetic consequences of cortical jaw deformities, functional impairments may also occur. These include ophthalmological problems such as exorbitism and respiratory problems such as obstructive sleep apnoea due to the compression by the expanding granulomas [2, 3].

SH3BP2 is an adaptor protein that is involved in bone remodelling and inflammation [10], interacting with various proteins such as Vav and Syk [11]. Cherubism mutations lead to a gain-of-function of SH3BP2. The mutated protein escapes from the tankyrase interaction, preventing the proteasome degradation of SH3BP2, which accumulates in the cytoplasm and increases that macrophage and osteoclast differentiation [10]. Although cherubism is typically described as an exclusive maxillofacial disease, the impact of *SH3BP2* mutations on bone remodelling and homeostasis has never been thoroughly investigated in the context of human pathology.

Experimental studies on a KI mouse model of cherubism, carrying *Sh3bp2* gain-of-function mutation, recently investigated the pathological mechanisms underlying the disease [12]. These studies demonstrated the autoinflammatory nature of the disease, showing the presence of systemic inflammation and bone loss in mutant mice [13]. Regarding human pathology, some authors have reported normal blood counts, and serum levels of electrolyte, calcium and phosphate, as well as normal levels of the hormones TSH, FSH, LH, PTH, PTHrP, T4 and T3, as well as calcitonin and osteocalcin [14, 15]. High levels of alkaline phosphatase have also been reported [6, 15, 16]. We recently presented a case study of a young female patient with cherubism associated and a low bone mass phenotype. Elevated levels of bone markers (CTx and P1NP) and inflammatory cytokines (IL-1β and TNFα) were also observed, suggesting that the cherubism phenotype may extend beyond the craniofacial region [17]. We then explored the systemic manifestations of cherubism in our patient group. Here, we present the systemic inflammatory and bone homeostasis phenotypes observed in our cohort of 11 patients with clinically and genetically confirmed cherubism. These are based on clinical data, markers of bone remodelling and inflammation, and bone densitometry.

## Materials and methods

### Patients

We recruited 11 patients via our reference centre, Maface, at Hôpital Necker – Enfants Malades). Written informed consent was obtained from all patients and their parents. These explorations were approved by the ethics committee at Necker Hospital.

### Positive cherubism diagnosis and evaluation of the extension of the lesions

Assessment of the cherubism phenotype was based on clinical and paraclinical data that were collected at the time of diagnosis or during subsequent examinations, depending on the patient. Radiological extension was evaluated using standard craniofacial CT scans and the cherubism severity was defined according to the Raposo-Amaral & Motamedi classifications [1, 4, 5]. Polysomnography was performed to evaluate the severity of functional consequences, in cases of clinical signs of obstructive sleep apnoea syndrome, as well as ophthalmological examinations in case of maxillary lesions.

### Confirmation of cherubism

To confirm the diagnosis of cherubism, the histopathological data of the jaw lesions were examined and genetic testing was performed. DNA samples were obtained from peripheral leucocytes. Direct bidirectional sequencing of exons and their flanking intron–exon boundaries was performed to screen for *SH3BP2* mutation. The entire coding region of *SH3BP2* was tested. Primers and PCR conditions are available on request.

The PCR products were sequenced using a Big Dye Terminator Cycle Sequencing Ready Reaction Kit (Applied Biosystems, Foster City, California, USA), and the reaction products were analyzed using an ABI 3100 Genetic Analyzer (Applied Biosystems). The sequences were aligned using Seqscape analysis software (Applied Biosystems) and compared with the reference sequences for genomic DNA. The GenBank accession number for genomic and mRNA sequence reference is *SH3BP2* NM_003023.4.

### Biological investigations

Standard biological investigations including blood and serum electrolytes tests and urine analysis, were performed according to conventional methods and adjusted for age.

Investigations of inflammation (fibrinogen, TNFα, IL-1, IL-6, IL-10, C-reactive protein (CRP), and white blood cell count) and bone remodelling markers (including the bone resorption marker, C-terminal telopeptide collagen crosslinks (Ctx), and the bone formation markers procollagen type I N-terminal propeptide (P1NP), bone alkaline phosphatase (BALP) and osteocalcin (OC)) were performed in the same centre. Other explorations included mineral metabolism (serum calcium and phosphate, 25-hydroxyvitamin D, 1,25-hydroxyvitamin D, parathyroid hormone (PTH), creatinine, and urinary calcium and phosphate). All of these data were adjusted for age, except for 25-hydroxyvitamin D and PTH which exhibit age-independent variations.

All the values obtained from the patient samples were compared with normal values according to age and sex (see supplemental information for details regarding the normal values).

### Bone densitometry

Areal bone mineral density (aBMD) was assessed for all patients in the same centre using dual-energy X-ray absorptiometry (DXA) with the Lunar Prodigy Advance DXA scanner (GE Lunar Corporation., Madison, Wisconsin, USA). aBMD z-scores were adjusted for height, age, and where necessary, for black ethnic origin using, the Lunar, paediatric reference curve adjusted for black ethnic origin.

### NFATc1 classification

Immunohistochemistry for NFATc1 was performed on paraffin sections from each patient’s granuloma as previously described. The localisation of NFATc1 in the multinucleated giant cells was used to define a three-grade scale ranging from low to high severity. A: no NFATc1 detection (low severity); B: NFATc1 detected only in the MGC cytoplasm (intermediate severity); and C: nuclear and cytoplasmic localisation of NFATc1 (severe cherubism) [18].

## Results

### Patients characteristics

Eleven patients were recruited from the MAFACE reference centre at Hôpital – Necker Enfants malades. The mean age of the patient group was 11.6 years old (± 1.2) (range 6–21 years old) and the mean age at diagnosis was 8.9 years old (± 1; range 4–15 years). The female-to-male ratio was 6/5 (54.5%/45.5%) (Table 1). Most of the patients had a familial cherubism (81%, *n* = 9/11 patients); two patients were half-brothers (P5 and P6) and two were sisters (P9 and P10). The causative mutation was identified in *SH3BP2* gene for all but one patient (P8), and have been previously reported. The most common mutation identified in this patient’s group was c.1244G > A (Table 1).

**Table 1:** Patient characteristics

Patient	Sex	Age de diag (year)	Age (year)	SH3BP2 mutation	Form	Radiological classification	Clinical symptoms	NFATc1 Classification	Height (cm)	Weight (kg)
P1	F	4	6	c. 1244 G > A	Hereditary	VI	Severe bilateral maxillary and mandibular swellings, nasal obstruction, OSA, exophtalmos	C	109,0	15,2
P2	M	6	9	c. 1244 G > A	Hereditary	VI	Severe bilateral maxillary and mandibular swellings, nasal obstruction, OSA, exophtalmos	C	139,0	35,0
P3	F	6	12	c. 1244 G > A	*de novo*	II.1	Maxillary and mandibular swellings	B	158,0	40,0
P4*	M	15	18	c. 1244 G > A	Hereditary	I.2	Unilateral mandibular swelling	A	174,5	72,8
P5*	M	7	10	c. 1244 G > A	Hereditary	II.1	Bilateral mandibular swellings	B	137,0	28,3
P6	F	7	7	c. 1244 G > A	Hereditary	I.5	Bilateral mandibular swellings	?	121,5	24,0
P7	M	12	21	not found	Hereditary	II.1	Bilateral mandibular swellings	B	180,0	68,5
P8	M	7	11	c. 1244 G > A	*de novo*	I.4	Unilateral mandibular swelling	A	136,0	29,0
P9**	F	14	14	c. 1244 G > A	Hereditary	I.2	Unilateral mandibular swelling	B	156,0	53,6
P10**	F	12	12	c. 1244 G > A	Hereditary	I.5	Bilateral mandibular swellings	B	161,0	58,6
P11	F	7,5	7,5	c. 1255 G > A	Hereditary	I.4	Unilateral mandibular swelling	B	123,0	23,1

* half-brothers, ** sisters

OSA: obstructive sleep apnoea

The radiological classification grades according to the Raposo-Amaral and Motamedi classifications [1, 4, 5] were heterogeneous, ranging from grade I (low), concerning six patients (54.5%) to grade VI (severe), concerning two patients (18.2%) (Table 1). The severity grade was also defined according to the NFATc1 location in the multinucleated giant cells (MGCs). Most of the patients were of grade B (54.5%, six out of eleven patients), two were of grade A (18,2%) and two were grade C (18.2%) [18].

**Fig. 1:**
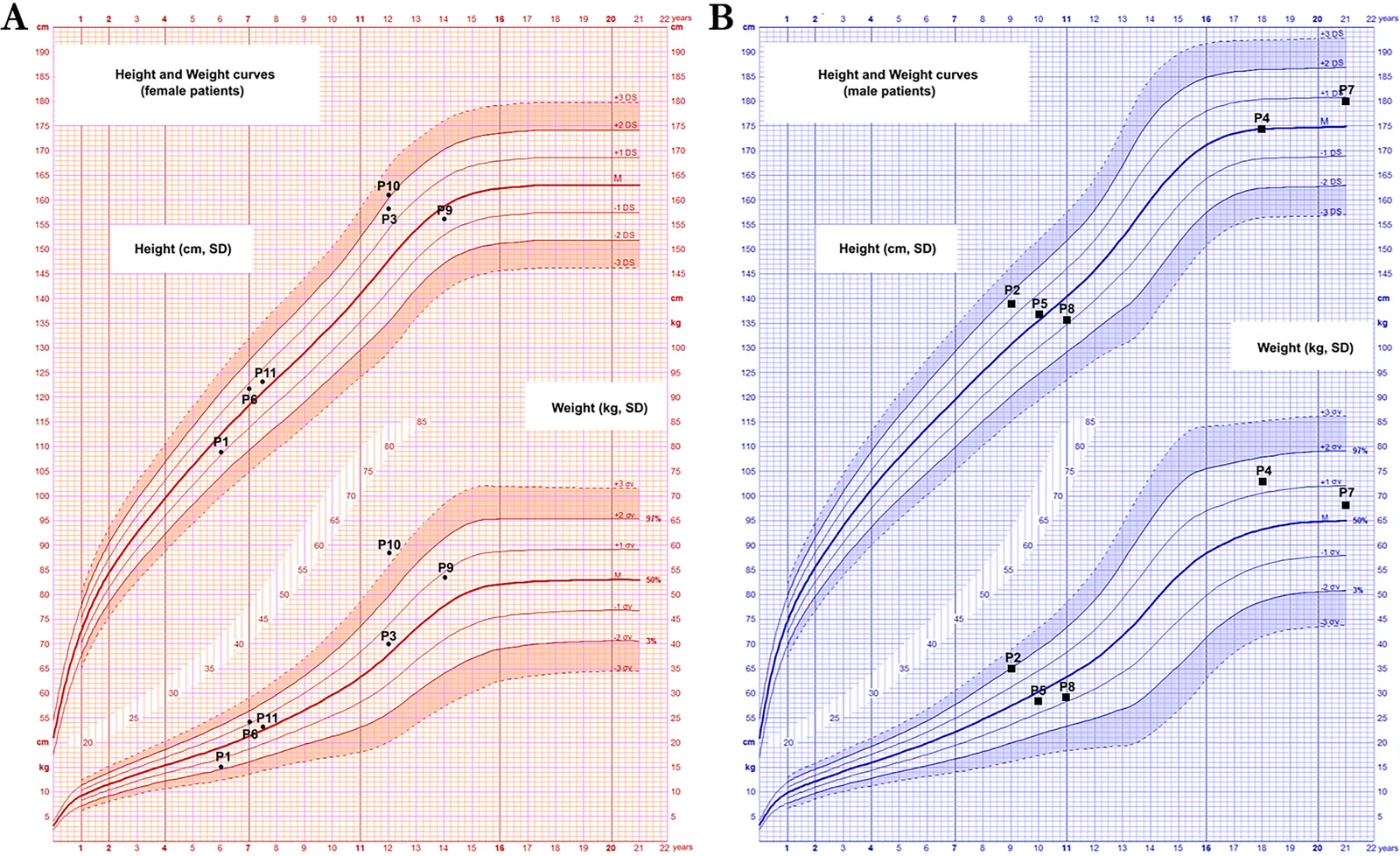
Growth curves for both size and weight showing the normal growth of the patients. **(A)** Female growth curves. **(B)** Male growth curves

Finally, stature-weight growth was evaluated. Weight and height were within the normal limits (Fig. 1A and B, and Table 1).

### Calcium phosphate metabolism markers

As cherubism is characterized by an osteolysis, the calcium phosphate metabolism markers (serum calcium and phosphate, 25-Hydroxyvitamin D, 1-25 diHydroxyvitamin D, Parathyroid Hormone (PTH), creatinin and urinary calium and phosphate) were explored in both serum and urine of the eleven patients, to identify any potential distrubances (Table 2). At the time of analysis, only a few patients had elevated parameters (Table 2). For two patients, the PTH level was elevated at the time of the surgery, but returned to normal quickly afterwards.

**Table 2:**
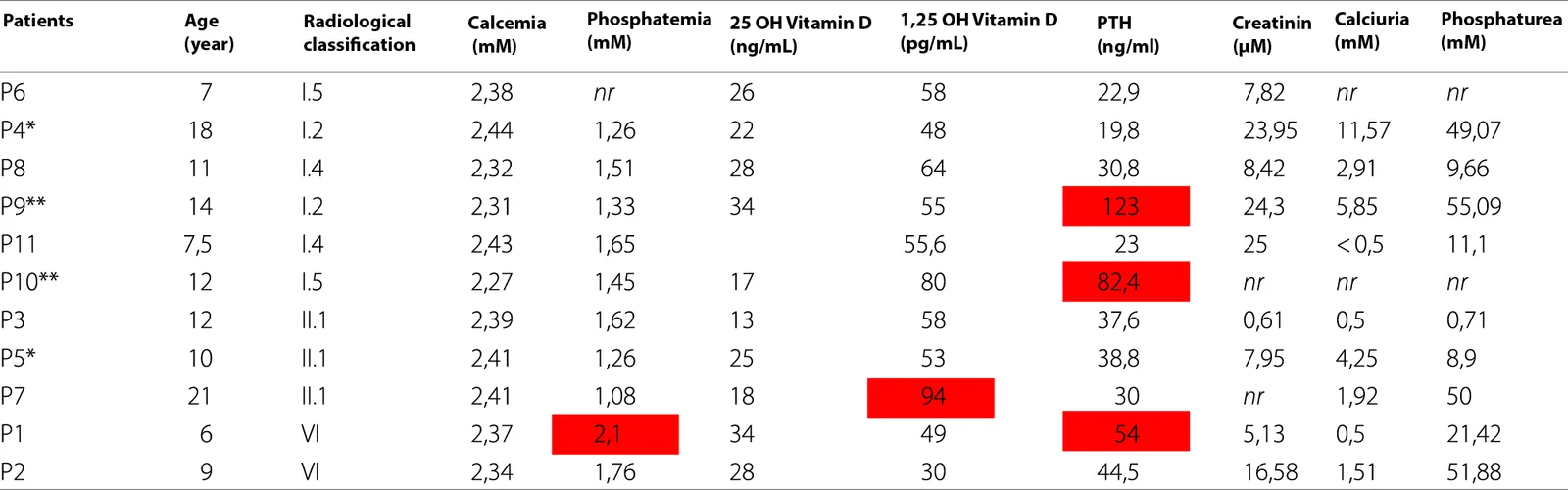
Patients’ blood levels of markers of calcium-phosphate metabolism

* half-brothers, ** sisters

Patients are organized according to their NFATc1 localization classification. The values highlighted in red are above the normal range for the patient’s age and sex

nr: not realized

### Inflammation markers

Cherubism has been described as an autoinflammatory condition [13]. Furthermore, Ueki et al. have described the central role of TNFα in the cherubism model [12]. A potential chronic inflammation has been explored in the serum of the eleven patients. TNFα levels were elevated in three patients (P1, P6 and P11), two of whom also had elevated IL-1β levels (P1 and P11). Two other patients (P6, and P8) had elevated IL-6 levels. CRP levels were within the normal range. Leucocyte counts were normal, though there was a slight elevation in the red blood cell count for four patients (Table 3).

**Table 3:**
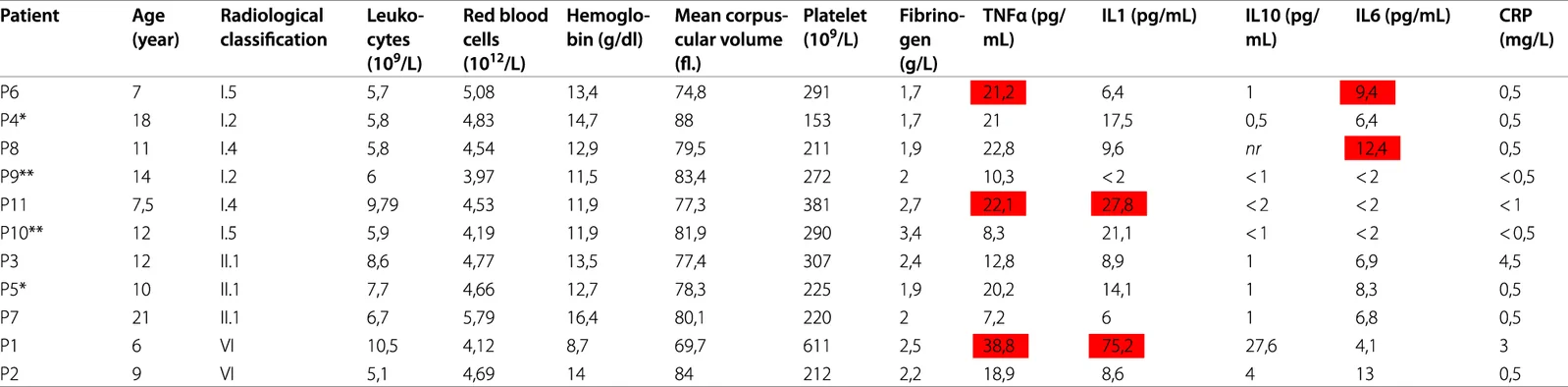
Patients’ blood counts and inflammatory marker levels

* half-brothers, ** sisters

Patients are organized according to their NFATc1 localization classification. The values highlighted in red are above the normal range for the patient’s age and sex

### Bone density

**Fig. 2:**
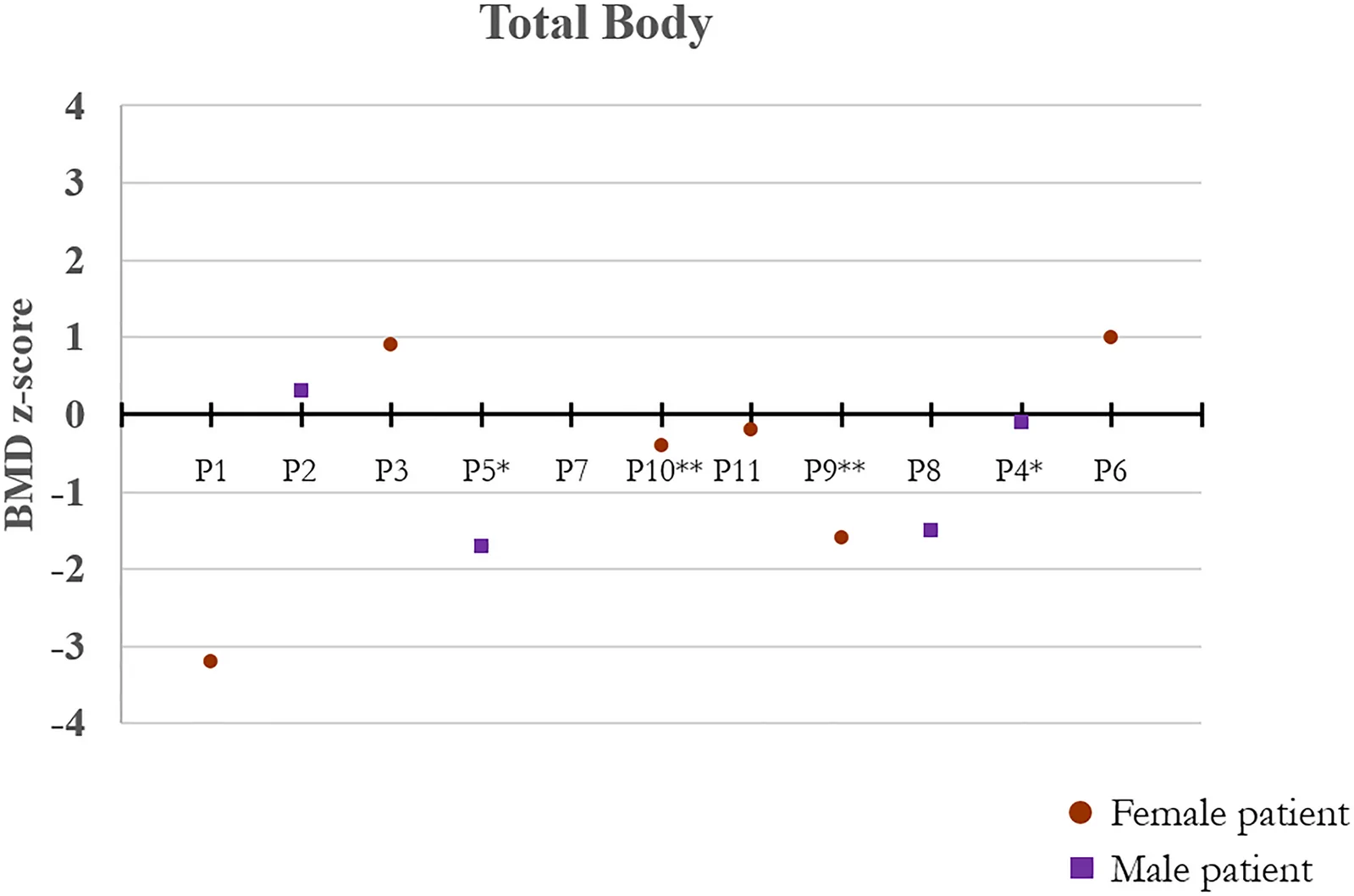
Bone density exploration. Graphical representation of the BMD z-score values for the total body according to the NFATc1 classification for each patient. Patients are ordered according to their radiological classification

As part of our exploration of the cherubism phenotype, we performed a DXA examination of each patient to analyze bone mineral density in the total body, femoral neck and lumbar spine. We previously reported that P1 had a low bone mass phenotype [17]. Of the eleven patients, eight presented low z-score values for at least one anatomical site, including five patients who were more severely affected and presented low z-score values for all the three sites (Fig. 2; Table 4).

**Table 4:**
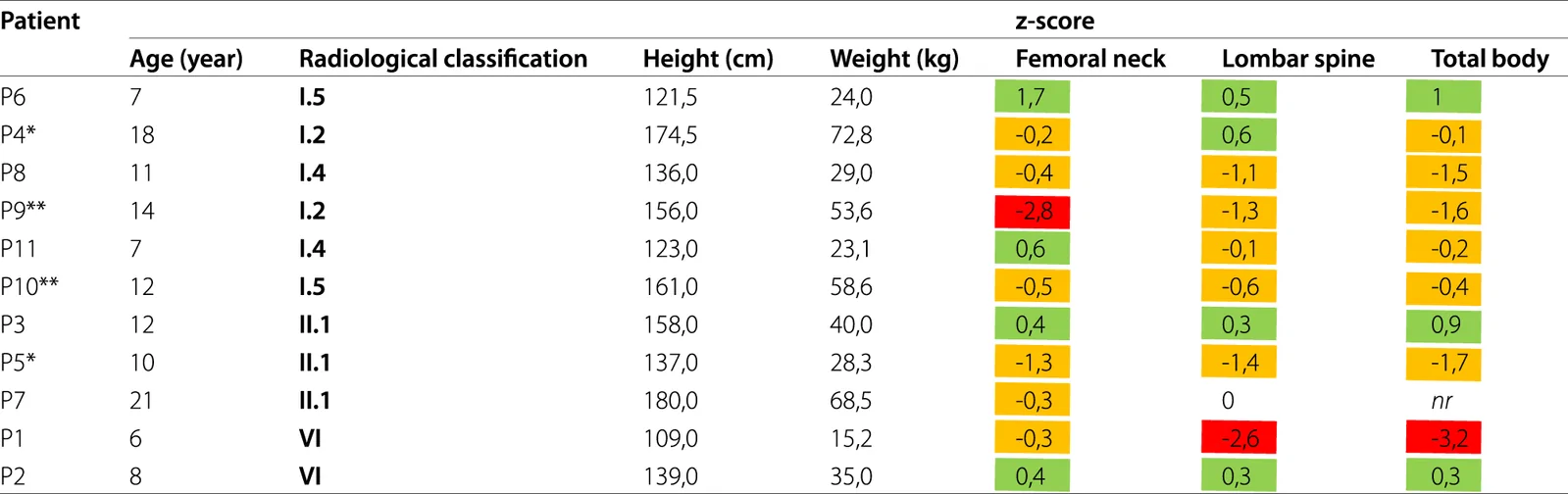
Patient aBMD z-score for total body, femur and lumbar sites

* half-brothers, ** sisters

Patients are organized according to their NFATc1 localization classification. Values in the upper part of the normal range are shown in green. Values in the lower part of the normal range are shown in orange. Values under the lower normal limit are shown in red

nr: not realized

### Bone metabolism markers

**Fig. 3:**
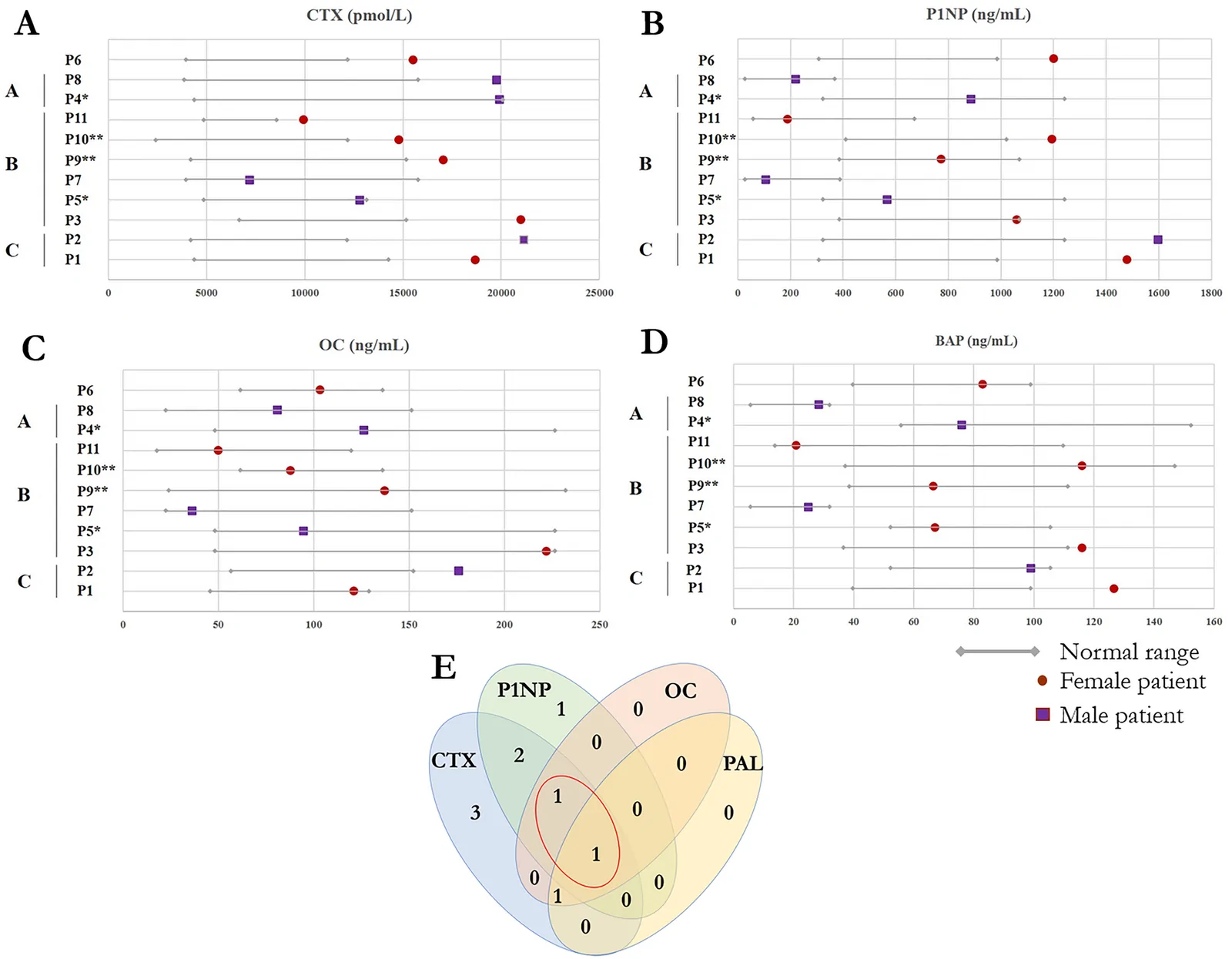
Bone metabolism markers according to the NFATc1 classification of the patient. For each patient, the bone marker value is represented graphically as a brown circle for female patients and a purple square for male patients. The normal ranges according to sex and age are represented in grey. **(A)** CTx values for each patient. **B**. P1NP values for each patient. **C**. Osteocalcin (OC) values for each patient. **D**. Bone alkaline phosphatase values for each patient. **E**. Venn diagram showing patients with three or four elevated bone metabolism markers

In addition to the DXA measurement, the bone metabolism blood markers were also explored (Table 5; Fig. 3). Levels of markers for bone resorption (CTx) and for bone formation (P1NP, OC and bone alkaline phosphatase) were analyzed in the serum of the eleven patients. Elevated CTx levels were found in eight patients (only P5, P7 and P8 were within the normal range). Elevated P1NP levels were found in four patients (P1, P2, P6 and P11). Bone alkaline phosphatase levels were elevated in two patients (P1 and P3). Osteocalcin levels were elevated in one patient (P2). Interestingly, three patients (P5, P7 and P8) had bone metabolism markers within the expected range for their age and sex. Three patients (P4, P9 and P10) had only one elevated marker, CTX. Patients P3, P6 and P11 had two elevated bone markers (CTX for all three, and elevated P1NP for P6 and P11, or BAL for P3). Patients P1 and P2 had three elevated markers (P1NP and CTX for both, and bone alkaline phosphatase for P1 and osteocalcin for P2). When the patients with elevated bone metabolism blood markers are represented in a Venn diagram, it appears that those with the most severe NFATc1 classification have 3 or 4 of such elevated markers (Fig. 3E).

**Table 5:**
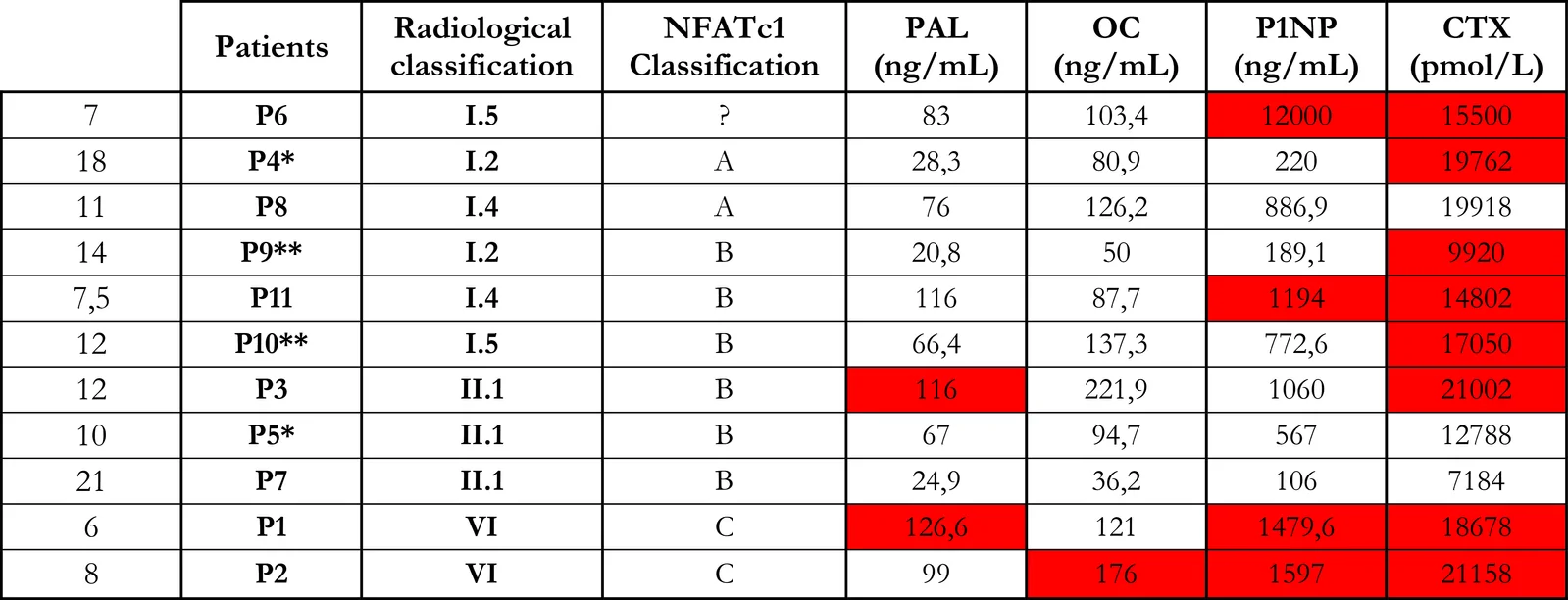
Patients’ bone metabolism blood marker levels

* half-brothers, ** sisters

The values above the normal range are shown in red, according to the sex and age of the patient. BAP: Bone Alkaline Phosphatase; OC: Osteocalcin; P1NP: Procollagen Type I N-Terminal Propeptide; CTx: Carboxy-Terminal Telopeptide Collagen Crosslinks

## Discussion

Although it was first described in 1933, cherubism remains a conundrum at many levels. Cherubism is an autosomal dominant rare bone disease associated with sometimes massive osteolysis of the jaws. Having recently described a generalized low bone mass phenotype associated with cherubism [17], we were prompted to explore a potential bone phenotype outside the craniofacial sphere. To this end, we recruited in our reference centre 11 patients with a confirmed diagnosis of cherubism.

The patient group that was explored was composed of six female and five male patients who were quite heterogeneous in terms of age, age of diagnosis, and radiological classification. To our knowledge, however, this is the first time that a group of cherubism patients has been explored systematically outside of the craniofacial sphere.

All patients except one (P6) underwent surgical curettage of the jawbone lesions with pathological examination at the time of initial diagnosis. One patient (P6) had a low radiological severity grade, with no morphological or functional impairment or tooth eruption disorder; thus, there was no indication for surgical intervention at this time.

Our exploration of a potential cherubism bone phenotype outside the craniofacial region shows that patients with cherubism have normal or subnormal growth in terms of stature and weight. However, our observations only relate to one timepoint: the time at which patients were included in the current study. These data must be confirmed in larger multicentre cohorts. Furthermore longitudinal growth analyses in patients with cherubism will help to clarify potential correlations between the natural progression of cherubism lesions in the jaws, inflammatory flare-ups and the growth of stature and weight over time.

Analysis of the calcium and phosphate metabolism did not reveal a clear profile of cherubism, even though three patients had elevated serum levels PTH, and nine patients had low levels of 25OH vitamin D. Vitamin D deficiency is frequently observed in the Caucasian population with low sun exposure, affecting around 60% of the population [19]. Regarding the inflammatory markers, only four patients had elevated serum levels of TNFα, IL1β and IL6. Elevated TNFα and IL1β serum levels were observed in two patients (P1 and P11) but there was no apparent association with the severity of their cherubism. This suggests that TNFα may not play as significant a role in human cherubism as it does in the mouse model [12]. No strict correlation was observed between the inflammatory phenotype and the radiological classification of cherubism lesions. DMO analysis revealed z-scores at the lower end of normal for most patients in our series (*n* = 8/11) for at least one anatomical site, with all sites affected in five patients. However, half of the patients with low z-scores (*n* = 4/8) had a Total Body z-score > -1.5 SD and were less severely affected. The most severely affected patient was Patient 1, who was previously reported [17] and presented with particularly severe clinical and biological phenotypes. It is hypothesized that the severe clinical, biological, and bone loss phenotypes observed in Patient 1 corresponded to an especially active period of cherubism characterized by giant cell granuloma proliferation and systemic inflammation. This hypothesis requires confirmation through multicentric, longitudinal studies.

Interestingly, the analysis of bone metabolism markers revealed elevated serum levels of the bone resorption marker CTx, and the bone formation markers P1NP, OC and BAL. Notably, such a precise anatomically localized disease as cherubism implies a significant elevation in CTx serum levels, indicating osteoclast activity and excessive bone resorption. Three patients presented with normal bone metabolism maker serum levels (P5, P7 and P8) and low grades of NFATc1 classification grades (A or B), which most probably reflect inactive or regressed phases of cherubism phases. This is consistent with the observation that the oldest patients in the study (P4 and P7, aged 18 and 21 years, respectively) exhibited less severe clinical and biological phenotypes in a disease characterized by the spontaneous regression of cherubism lesions activity with age. These observations, based on the analyses of variations of bone metabolism markers strongly suggest that cherubism patients should undergo regularly clinical and biological monitoring to gain insight into the evolution of the disease. In particular, longitudinal analyses of clinical and biological phenotypes may reveal potential predictive paraclinical parameters of cherubism evolution, that could inform the therapeutic decisions and improve patient care. This study suggests an association between the radiographic severity, the NFATc1 severity and the elevated bone metabolism blood markers. However, these observations were only made at one time point. A longitudinal and if possible multicentre study should be carried out. In addition, some other severity markers should also be identified. These potential predictive parameters could be of particular interest in helping to decide on systemic treatments (type and duration) [20, 21] in severe, clinically active forms with life-threatening giant cell granuloma extension, and in evaluating treatment response and efficacy. A better understanding of the natural progression of cherubism and how to predict it may also inform decisions regarding conservative and non-surgical treatments in less severe cases, when parameters indicate ongoing disease regression.

Our analyses of radiological extensions [4, 5] also suggest that the grades defined at initial diagnosis should be also re-evaluated during the follow-up. For example, P6 initially exhibited a low grade of radiological extension (at 7 years of age), but by the time of the present study (16 years of age), she had developed a severe grade (VI) of granuloma jaw extension, indicating the need for surgical curettage. The radiological classifications used for cherubism jaw lesions are indeed based on the location and the number of osteolytic lesions but they may not reflect the current activity of the disease or its systemic repercussions. Our study shows that MGCs nuclear NFATc1 localization (grade C) is associated with more severe forms of cherubism, as observed in P1 and P2. These patients presented with more severe radiological grades and elevated levels of both bone formation and remodeling markers, as well as low bone density in the patient with the most severe form of cherubism in this group (P1). The second most severe patient (P2) did not present low bone density at the time of the study. However, the causative factors with low bone density are multifactorial, and include genetic and environmental factors. Bone density may also vary with age and during growth. Furthermore, low bone density may manifest with a delay following the onset of jawbone lesions in cherubism. It is worth noting that P1 was Gabonese and was not managed at our centre immediately after the onset of the disease, but only at an extremely severe stage of the disease. Bone density was assessed at only one timepoint in the present study, at the initial stage of the patient’s treatment at our centre. A longitudinal evaluation of bone density evolution could provide new insights into the natural progression of cherubism in terms of inflammation and bone homeostasis disruption.

Generating mice models of cherubism helped to decipher the underlying molecular mechanisms that lead to the development of giant cell granulomas and excessive bone resorption. These knock-in mouse models express the P416R [12] or G418A [22] mutation, which is equivalent to the human *SH3BP2* P418R or G420A mutation [12, 22]. It has been demonstrated that an activating mutation in *Sh3bp2* increases the NFATc1 nuclear translocation downstream of RANK-L induced PLCγ phosphorylation. This leads to increased induction of osteoclast-specific genes and formation of multinuclear giant cell [23].

Cherubism is classified as an autoinflammatory disease [24]. However, the mechanisms that stimulate this process are not fully understood. Several hypotheses based on studies of the mouse models have been proposed to explain the initiation of the autoinflammatory response. Among these hypotheses is the idea that toll-like receptors (TLR) are involved in response to changes in oral microbiota changes or damage-associated molecular patterns during jaw remodelling [22, 24]. Furthermore, these *Sh3bp2*^*ki/ki*^ mouse models exhibit systemic bone loss and granulomas that are not confined to the craniofacial region. Therefore, mice models of cherubism do not fully mimic human pathology. While *Sh3bp2*^*ki/ki*^ mice model exhibit severe osteopenia, *Sh3bp2*^*ki/+*^ mice only exhibit mild osteopenia and no inflammation [12].

Studies have shown that heterozygous mice acquire a phenotype close to that of homozygous mice when under the influence of inflammatory stimulations [12]. This stimulus, which is necessary for the initiation or activation of a genetically determined pathology, corresponds to human pathology, which exhibits spatial (maxilla-mandible) and time (2–15 years) determinism. Extrapolating this, we could envisage that tooth eruption being responsible for this activation through multiple mechanisms, leading to changes in the microenvironment favourable to the development of cherubism, as in heterozygous mice.

This study is the first to explore a cherubism phenotype outside the craniofacial region in a group of eleven patients. While this is an important cohort given the rare characteristics of cherubism, further observations are needed in a larger number of patients at different centers. We recommend monitoring the growth curves of the patients as the diseases progresses. We also recommend monitoring bone mineral density and bone and inflammation markers thorough the course of the disease. Overall, our findings highlight that cherubism is an evolving disease and that systematic longitudinal clinical and paraclinical investigations could help to better characterize its natural evolution and systemic repercussions more accurately, thereby improving patient care and understanding of the disease.

## Supplementary Information

Below is the link to the electronic supplementary material.

Supplementary Material 1

## Data Availability

All the data supporting our findings are contained within the manuscript.
