# Supplementary material for: Exploring a cherubism bone phenotype outside the craniofacial region
Source: Orphanet J Rare Dis. 2026 Jun 11;21:290. doi: 10.1186/s13023-026-04413-3 (PMC13487941; doi:10.1186/s13023-026-04413-3)
Supplement: Supplementary file 1 [file 13023_2026_4413_MOESM1_ESM.docx]

**Supplemental tables**

**Supplemental Table 1 : Cherubism radiological grading system adapted from Kalantar Motamedi and Raposo-Amaral** [1,2]

| **Grade I**  **Lesion of the mandible without signs of root resorption** | **Class 1 Solitary lesion of the mandibular body** |
| --- | --- |
|  | **Class 2 Multiple lesions of the mandibular body** |
|  | **Class 3 Solitary lesion of the ramus** |
|  | **Class 4 Multiple lesions of the rami** |
|  | **Class 5 Lesions of the mandibular body and rami** |
| **Grade II**  **Lesions involving the mandible and maxilla without sign of root resorption** | **Class 1 Mandibular and maxillary tuberosity** |
|  | **Class 2 Mandible and anterior maxilla** |
|  | **Class 3Mandinble and whole maxilla** |
| **Grade III**  **Aggressive lesions of the mandible with signs of root resorption** | **Class 1 Solitary lesion of the mandibular body** |
|  | **Class 2 Multiple lesions of the mandibular body** |
|  | **Class 3 Solitary lesion of the ramus** |
|  | **Class 4 Multiple lesions of the rami** |
|  | **Class 5 Lesions of the mandibular body and rami** |
| **Grade IV**  **Lesions involving both the mandible and maxilla and showing signs of root resorption** | **Class 1 Mandibular and maxillary tuberosity** |
|  | **Class 2 Mandible and anterior maxilla** |
|  | **Class 3Mandinble and whole maxilla** |
| **Grade V**  **The rare, massively growing, aggressive and extensively deforming juvenile cases involving the mandible and maxilla and may include the coronoid and condyles** | |
| **Grade VI**  **The rare, massively growing, aggressive and extensively deforming juvenile lesions involving the mandible, maxilla and orbits** | |

**Supplemental Table 2 : Normal values 1**

| **Name** | **Lower values** | **Upper values** | **Units** |
| --- | --- | --- | --- |
| **Fibronogen** | 1,5 | 3,5 | g/L |
| **Creatinin** | 62 | 106 | µM |
| **Calcemia** | 2,2 | 2,7 | mM |
| **Phosphatemia** | 1,3 | 1,85 | mM |
| **25 OH** | 30 | 60 | ng/mL |
| **PTH** | 10 | 50 | ng/ml |
| **Creatinin** | 62 | 106 | µM |
| **Calciuria** | ? | ? | ? |
| **Phosphateria** | ? | ? | ? |
| **TNF** α | 0 | 20 | pg/mL |
| **IL-1β** | 0 | 15 | pg/mL |
| **IL-10** | 0 | 112 | pg/mL |
| **IL-6** | 0 | 8,6 | pg/mL |
| **CRP** | <6 |  |  |

**Supplemental Table 3 : Normal values 2**

| **Name** | **Children** | | **Adults** | | **Units** |
| --- | --- | --- | --- | --- | --- |
|  | **Lower values** | **Upper values** | **Lower values** | **Upper values** |  |
| **Leucocytes** | 5,5 | 15,5 | 4 | 10 | 10^9^/L |
| **Red Blood cells** | 3,9 | 4,6 | 4,5 | 6,2 | 10^12^/L |
| **Hemoglobin** | 11,5 | 13,5 | 13 | 18 | g/dl |
| **Mean corpuscular volume** | 77 | 86 | 83 | 98 | fl |
| **Platelets** | 175 | 420 | 150 | 450 | 10^9^/L |

**Supplemental Table 4 : 1,25 OH normal values** [3]

| **Age** | **pg/mL** |
| --- | --- |
| **0 – 1 year** | 32 – 198 |
| **1 – 3 years** | 47.5 – 152.5 |
| **Children (<11 years)** | 30 – 80 |
| **Adolescents (11 – 16 years)** | 30 – 120 |
| **Adults** | 29 – 83.6 |

**Table 5 : Osteocalcin normal values** [4,5]

| **Girls (ng/mL)** | | **Boys (ng/mL)** | |
| --- | --- | --- | --- |
| **6** **months** **–** **1** **year** | 44.9 – 122.5 | **6** **months** **–** **1** **year** | 45.1 – 116.5 |
| **1** ye**ar** | 38.9 – 121.5 | **1** year | 45.6 – 124.5 |
| **2** **years** | 44.9 - 116 | **2** **years** | 37.8 – 120.3 |
| **3** **years** | 40.6 – 114.6 | **3** **years** | 38.1 – 105.7 |
| **4** **years** | 44.0 – 122.2 | **4** **years** | 39.0 – 112.9 |
| **5** **years** | 45.4 – 118.6 | **5** **years** | 44.3 – 116.9 |
| **6** **ans** | 45.8 – 128.9 | **6** **years** | 42.3 – 112.0 |
| **6** **–** **10** **years** | 61.4 – 136.2 | **6** **–** **9** **years** | 56.5 – 152.1 |
| **10** **–** **14** **years** | 24.1 – 232.1 | **9** **–** **15** **years** | 48.2 – 226.4 |
| **14** **–** **15** **years** | 17.8 – 119.6 |  |  |
| **>** **15** **years** | 21.1 – 76.7 | **>** **15** years | 22.5 – 151.3 |

| **Women (ng/mL)** | | **Men (ng/mL)** | |
| --- | --- | --- | --- |
| **20 – 30 years** | 26.6 - 51 | **20 – 30 years** | 24 - 70 |
| **>30 years** | 11 - 43 | **30 – 55 years** | 14 - 42 |
| **>50 years** | 15 - 46 | **>55 years** | 14 – 46 |

**Table 6 : Cross laps (CTX) normal values** [4,6,7]

| **Age** | **Girls (pmol/L)** | **Boys (pmol/L)** |
| --- | --- | --- |
| **6 months – 1 year** | 3177 - 13872 | 3487 - 12632 |
| **1 years** | 3410 - 12400 | 4572 - 13252 |
| **2 years** | 4185 - 13640 | 4107 - 13175 |
| **3 years** | 4262 - 13795 | 4030 - 13252 |
| **4 years** | 4882 - 13950 | 3952 - 13175 |
| **5 years** | 3797 - 14570 | 5115 - 13795 |
| **6 years** | 3875 - 14260 | 4185 - 13020 |
| **6 – 9 years** | 4379 – 12167 | 3945 – 13152 |
| **10 – 13 years** | 4859 – 15151 | 6665 – 20080 |
| **14 – 18 years** | 2418 - 8556 | 4185 - 15771 |

**Supplemental Table 7 : Procollagen 1 N-Terminal Propeptide (P1NP) normal values** [7–9]

| **Age** | **Girls (ng/mL)** | **Boys (ng/mL)** |
| --- | --- | --- |
| **< 4 years** | 174 - 1079 | 299 - 1010 |
| **5 – 8 years** | 307 - 985 | 200 - 900 |
| **9 – 12 years** | 386 - 1070 | 323 - 1242 |
| **13 – 16 years** | 59 - 672 | 142 - 6929 |
| **17 – 20 years** | 25 - 160 | 28 - 369 |

| **Women (ng/mL)** | | **Men (ng/mL)** | |
| --- | --- | --- | --- |
| **< 30 years** | 25 - 90 | 25 – 70 years | 15 - 80 |
| **30 – 39 years** | 15 - 80 |  |  |
| **40 – 49 years** | 15 - 60 |  |  |
| **50 – 69 ans** | 15 - 75 |  |  |

**Supplemental Table 8 : Bone alkaline phosphatase normal values** [4,10]

| **Age** | **Girls ng/mL** | **Boys ng/mL** |
| --- | --- | --- |
| **6-11 mois** | 37.6 – 118.6 | 35.9- 131.3 |
| **1 – 2 years** | 32.8 – 137.6 | 31.0 – 177.7 |
| **2- 3 years** | 31.9 – 106.4 | 28.3 – 104.4 |
| **3 – 4 years** | 32.8 – 105.5 | 29.5 – 106.4 |
| **4 – 5 years** | 28.4 – 107.2 | 37.2 – 106.6 |
| **5 – 6 years** | 39.6 – 98.9 | 35.3 – 99.6 |
| **7 – 8 years** | 37.1 – 147.9 | 52.6 – 123.0 |
| **9 – 10 years** | 42.0 – 107.6 | 52.3 – 105.4 |
| **11 – 12 years** | 38.6 – 111.2 | 55.7 – 152.3 |
| **13 – 14 years** | 13.7 – 109.8 | 15.5 – 134.0 |
| **15 – 16 years** | 10.2 – 72.6 | 16.6 – 127.9 |
| **17-18 years** | 5.9 – 20.0 | 11.0 – 77.6 |
| **>18 years** | 4.7 – 27.0 | 5.7 - 32 |
| **After menopause** | 5.5 – 27.0 | *na* |

**Supplemental references**

1. Kalantar Motamedi MH. Treatment of cherubism with locally aggressive behavior presenting in adulthood: report of four cases and a proposed new grading system. J Oral Maxillofac Surg. 1998;56:1336–42.

2. Raposo-Amaral CE, de Campos Guidi M, Warren SM, Almeida AB, Amstalden EMI, Tiziane V, et al. Two-stage surgical treatment of severe cherubism. Ann Plast Surg. 2007;58:645–51.

3. Higgins V, Truong D, White-Al Habeeb NMA, Fung AWS, Hoffman B, Adeli K. Pediatric reference intervals for 1,25-dihydroxyvitamin D using the DiaSorin LIAISON XL assay in the healthy CALIPER cohort. Clin Chem Lab Med. 2018;56:964–72.

4. Wyness SP, Roberts WL, Straseski JA. Pediatric reference intervals for four serum bone markers using two automated immunoassays. Clin Chim Acta. 2013;415:169–72.

5. Huang Y, Eapen E, Steele S, Grey V. Establishment of reference intervals for bone markers in children and adolescents. Clinical Biochemistry. 2011;44:771–8.

6. Melo VCP de, Ferreira PRS, Ricardi LO, Batista MC, França CN, Ferreira CE dos S. Definition of reference ranges for β-isomerized carboxy-terminal telopeptide collagen type I for children and adolescents. Journal of Pediatric Endocrinology and Metabolism. 2018;31:637–40.

7. Jenkins N, Black M, Paul E, Pasco JA, Kotowicz MA, Schneider H-G. Age-related reference intervals for bone turnover markers from an Australian reference population. Bone. 2013;55:271–6.

8. Morovat A, Catchpole A, Meurisse A, Carlisi A, Bekaert A-C, Rousselle O, et al. IDS iSYS automated intact procollagen-1-N-terminus pro-peptide assay: method evaluation and reference intervals in adults and children. Clin Chem Lab Med. 2013;51:2009–18.

9. Vasikaran SD, Miura M, Pikner R, Bhattoa HP, Cavalier E, IOF-IFCC Joint Committee on Bone Metabolism (C-BM). Practical Considerations for the Clinical Application of Bone Turnover Markers in Osteoporosis. Calcif Tissue Int. 2023;112:148–57.

10. Cavalier E, Rozet E, Carlisi A, Bekaert A-C, Rousselle O, Hubert P, et al. Analytical validation of serum bone alkaline phosphatase (BAP OSTASE) on Liaison. Clin Chem Lab Med. 2010;48:67–72.
